# Furosemide and spironolactone doses and hyponatremia in patients with heart failure

**DOI:** 10.1186/s40360-020-00431-4

**Published:** 2020-08-03

**Authors:** Ivan Velat, Željko Bušić, Marina Jurić Paić, Viktor Čulić

**Affiliations:** 1 University Hospital Center Split, Split, Croatia; 2 Department of Cardiology, University Hospital Center Split, Šoltanska 1, 21000 Split, Croatia; 3 University of Split School of Medicine, Split, Croatia

**Keywords:** Hyponatremia, Heart failure, Diuretics, Furosemide, Spironolactone

## Abstract

**Background:**

Hyponatremia, a marker of disease severity and prognosis, has been associated with various clinical factors and drug use, especially diuretics.

**Methods:**

This observational prospective cohort study enrolled patients hospitalized at the University Hospital Center Split because of heart failure (HF). We investigated the association of clinical variables and cardiovascular drugs, including furosemide, hydrochlorothiazide, spironolactone, and their doses, with the presence of hyponatremia at admission.

**Results:**

Of the 565 included patients, 32.4% were hyponatremic, 62.6% were males, and the mean age was 73.1 ± 10.6 years. In the univariate analysis, hyponatremic patients were more often current smokers (*p* = 0.01), alcohol consumers (*p* = 0.01), receiving spironolactone (*p* = 0.004) or combination of furosemide and spironolactone (*p* = 0.003). Patients who received 50 and 100 mg of spironolactone, compared to those receiving 25 mg (*p* < 0.0001), as well as patients who received 250 to 500 mg of furosemide compared to ≤240 mg (*p* = 0.001), were significantly more often hyponatremic. In the multivariate analysis, when diuretic doses were accounted for, furosemide doses of 250 to 500 mg (*p* = 0.009), spironolactone doses of 50 to 100 mg (*p* = 0.0003), increasing age (*p* = 0.03), diabetes mellitus (*p* = 0.02) and alcohol consumption (*p* = 0.04) were independently associated with hyponatremia.

**Conclusion:**

High doses of furosemide and spironolactone, or concomitant use of these diuretics, seem to be an important cause of hyponatremia in HF patients, particularly in combination with advanced age, diabetes and alcohol consumption. Diuretic dose reduction may help avoid hyponatremia and improve clinical status and prognosis in such patients.

## Background

Hyponatremia (a low serum sodium level) is the most common electrolyte disorder observed in hospitalized patients [1–3], with increased prevalence in the elderly [4, 5]. At the same time, hyponatremia has been established as an important marker of disease severity and prognosis [1, 2, 6]. Several clinical conditions and cardiovascular risk factors, such as diabetes mellitus [7, 8], chronic kidney disease [9], chronic obstructive pulmonary disease [10], cirrhosis [6] and alcohol consumption [11], may also be associated with hyponatremia.

Although multiple neurohormonal mechanisms are involved in its pathogenesis, hyponatremia can also be a consequence of drugs, most notably of diuretic therapy [9, 12, 13]. This therapy is prescribed to patients with chronic renal failure [14], arterial hypertension [14], cirrhosis [6], and especially to those with symptomatic heart failure (HF) [9]. Thiazides, loop diuretics, and potassium sparing diuretics induce water and salt excretion [12, 14, 15] and may promote sodium loss through renal effects [16]. It has been suggested that some diuretic combinations may additionally increase the risk of hyponatremia [17, 18].

The aim of the present study was to explore the associations of cardiovascular drug use, particularly three most commonly used diuretics in Croatia, i.e. furosemide, hydrochlorothiazide and spironolactone, with hyponatremia in patients with HF. We additionally aimed to assess the possible role of diuretic drug doses and drug coadministration in the occurrence of hyponatremia in this clinical setting.

## Methods

We performed an observational prospective cohort study at the Department of Cardiology and Department of Internal Medicine of the University Hospital Center Split, between February 1, 2015 and September 1, 2017. The study population included patients hospitalized because of HF (first manifestation or exacerbation of an existing disease). The inclusion criteria were: 1) clinical presentation typical for HF; 2) left ventricular ejection fraction (LVEF) ≤45% assessed by the Simpson method and/or diastolic dysfunction determined by echocardiography and 3) unchanged medications, including diuretic doses, for at least 1 month prior to hospital admission. Patients were excluded from the study if they had any acute or chronic disease other than elements of cardiorenal syndrome, that could disturb serum sodium levels (i.e., acute or chronic infections, endocrine and metabolic disorders, autoimmune, malignant, obstructive pulmonary or primary liver diseases).

For each patient, a structured questionnaire was filled out by specially trained interns or medical students, containing questions on general data, presence of previous cardiovascular diseases, cardiovascular risk factors and prehospital drug use. Because we were particularly interested in the effect of dose of the three most commonly used diuretics in Croatia, we collected data on the exact dose of furosemide (between 20 and 500 mg), hydrochlorothiazide (12.5 or 25 mg) and spironolactone (25, 50 or 100 mg). A small number of patients with loop diuretic torasemide (*n* = 5) and potassium sparing diuretic eplerenone (*n* = 14) were excluded from the analysis. In Croatia, triamterene and amiloride are only exceptionally used and we had no patients who used these drugs. Other cardiovascular drugs were included in the analyses as dichotomous variables.

Basic heart rhythm was determined by electrocardiogram at admission. Blood samples were taken within the first hour of hospitalization. Initial electrolyte levels were determined as a part of the normal diagnostic evaluation. Hyponatremia was defined as serum sodium level < 136 mmol/L. Elevated blood pressure (≥130/85 mmHg) or use of antihypertensive medication were criteria for hypertension. Elevated nonfasting glucose (≥11.1 mmol/L) or previously established diabetes mellitus were criteria for diabetes diagnosis. Plasma concentration of N-terminal pro brain natriuretic peptide (NT-proBNP), in pmol/L, was measured by using immunoassays (Roche Diagnostics GmbH, Mannheim, Germany). Kidney failure was defined as an estimated glomerular filtration rate (GFR) < 60 mL/min/1.73 m^2^, calculated using the 4-variable Modification of Diet in Renal Disease formula [19]. All patients underwent standard transthoracic echocardiographic examination at rest.

### Statistical analysis

The data were expressed as percentages for dichotomous variables. Normally distributed continuous variables were presented as means with standard deviation. Variables with a skewed distribution were expressed as medians with interquartile ranges (IQR). Associations of clinical variables with hyponatremia were tested using *χ*^*2*^ test, *χ*^*2*^ goodness-of-fit test, Student *t*-test and Mann-Whitney-*U* test, as appropriate. Correlations among continuous clinical variables were tested by linear regression analysis and expressed through the Pearson’s correlation coefficient. In the multivariate analysis, a stepwise logistic regression was used to test the predictability of occurrence of hyponatremia in terms of baseline characteristics, LVEF, cardiovascular risk factors, and drug use, including diuretic doses in three different models. A multiple regression analysis (with calculation of the standardized correlation coefficient) was used to estimate the independent predictive association of baseline clinical characteristics, other markers of HF severity and cardiovascular drugs with serum NT-proBNP levels. A *p* value of < 0.05 was considered statistically significant. All statistical analyses were performed using IBM Statistical package for the social science (SPSS), software version 20.0 (SPSS Inc., Chicago, Illinois).

## Results

Table 1 shows baseline characteristics of the 565 included patients. Average sodium level for all patients was 137.7 ± 4.4 mmol/L (133.1 ± 4.1 mmol/L for hyponatremic and 139.9 ± 2.3 mmol/L for non-hyponatremic patients). There were 41.1% patients with LVEF ≤45% (Table 1). Out of these patients, 35.1% had a mild dysfunction (LVEF 40–45%), 38.8% had moderate (LVEF 30–39%), and 26.1% (LVEF < 30%) had severe dysfunction. Compared to non-hyponatremic patients, hyponatremic patients were more often current smokers or alcohol consumers, more often had a LVEF ≤45%, and less often had atrial fibrillation or flutter (Table 1). Hyponatremic patients on average had a higher urea and serum potassium, but lower serum chloride (Table 2).

**Table 1:** Prehospital drugs and clinical characteristics of the study population and in study patients according to hyponatremia (*N* = 565)

Clinical characteristics	All patients	Hyponatremic patients (*N* = 183)	Non-hyponatremic patients (*N* = 382)	*p*
Age (years ± SD)	73.1 ± 10.6	74.3 ± 9.4	72.9 ± 10.5	0.15
Male gender (%)	62.6	62.3	63.4	0.81
LVEF ≤45% (%)	41.1	48.1	37.7	0.02*
Previous AMI (%)	20.5	24.6	18.6	0.10
Arterial hypertension (%)	66.9	63.4	68.6	0.22
Diabetes mellitus (%)	33.8	38.8	34.4	0.08
Kidney failure (%)	28.2	31.7	26.4	0.19
Alcohol consumption (%)	30.3	33.3	23.8	0.01*
Current smokers (%)	14.5	20.2	11.8	0.01*
Atrial fibrillation/ flutter (%)	49.5	41.5	53.4	0.008*
Prehospital medication (%)				
Furosemide	60.3	64.5	58.4	0.17
Hydrochlorothiazide	16.5	14.8	17.2	0.45
Spironolactone	17.9	24.6	14.7	0.004*
β-blocker	55.4	51.9	57.1	0.25
Calcium antagonist	20.5	19.1	21.2	0.14
ARB	10.9	13.1	9.9	0.26
ACEI	40.7	39.3	41.4	0.65
Aspirin	31.1	27.9	32.7	0.24
Digoxin	22.5	25.1	21.2	0.30
Coadministration of diuretics (%)^a^				
Furosemide + spironolactone	16.5	23	13.1	0.003*
Furosemide + hydrochlorothiazide	6.2	7.7	5.8	0.39
Furosemide + hydrochlorothiazide + spironolactone	1.9	2.2	1.8	0.78

Hyponatremia: serum sodium < 136 mmol/L, *LVEF*: left ventricular ejection fraction, *AMI*: acute myocardial infarction, *ARB* Angiotensin II receptor I blocker, *ACEI* Angiotensin converting enzyme-inhibitor

*p* values refer to the *t*-test or *χ*^*2*^ test as appropriate

*Statistically significant differences (*p* < 0.05)

^a^There were no patients with hydrochlorothiazide + spironolactone combination

**Table 2:** Laboratory findings of the study patients according to hyponatremia (*N* = 565)

Laboratory findings	Hyponatremic patients (*N* = 183)	Non-hyponatremic patients (*N* = 382)	*p*
Hemoglobin (median, IQR, g/L)	127 (112–144)	132 (116–144)	0.57
Hematocrit (mean ± SD, L/L)	0.39 ± 0.07	0.4 ± 0.07	0.11
Urea (median, IQR,mmol/L)	10.5 (7–15.5)	8.7 (6.3–12.2)	0.004*
Serum creatinine (median, IQR, mg/dL)	1.4 (1.1–1.9)	1.3 (1–1.7)	0.20
Serum potassium (mean ± SD, mmol/L)	4.3 ± 0.7	4.1 ± 0.6	0.001*
Serum chloride (mean ± SD, mmol/L)	97.2 ± 5.5	101.2 ± 4.4	< 0.001*
Cardiac troponin I (median, IQR, ng/ml)	0.051 (0.020–0.108)	0.039 (0.018–0.089)	0.12
Total cholesterol (median, IQR, mmol/L)	3.4 (3.1–4.9)	4.3 (3.5–5)	0.57
HDL (median, IQR, mmol/L)	1.3 (0.9–1.8)	1.3 (1.3–1.8)	0.71
LDL (median, IQR, mmol/L)	2.4 (1.9–3.2)	2.5 (1.9–3.1)	0.92
Triglycerides (median, IQR, mmol/L)	1.8 (1.4–2.5)	1.8 (1.2–2.4)	0.44
Total bilirubin (median, IQR, μmol/L)	15.9 (11.0–21.0)	14.3 (11.2–18.9)	0.26
Direct bilirubin (median, IQR, μmol/L)	4.0 (2.4–7.0)	4.0 (2.8–6.0)	0.68
Indirect bilirubin (median, IQR, μmol/L)	11.0 (8.6–13.9)	10.2 (8.1–13.1)	0.08
Serum uric acid (median, IQR, μmol/L)	476 (381–574)	440 (367–545)	0.10
AST (median, IQR, μmol/L)	27.0 (19.0–37.0)	24.0 (19.0–33.0)	0.12
ALT (median, IQR, μmol/L)	23.0 (15.8–33.0)	23.0 (17.0–34.3)	0.68
GGT (median, IQR, μmol/L)	45.0 (25.0–97.0)	39.0 (23.0–82.0)	0.16

Hyponatremia: serum sodium < 136 mmol/L, *HDL* High-density lipoprotein, *LDL* Low-density lipoprotein, *AST* Aspartate aminotransferase, *ALT* Alanine aminotransferase, *GGT* Gamma-glutamyl transferase

*p* values refer to the Mann-Whitney *U* test and *t*-test as appropriate

* Statistically significant differences (*p* < 0.05)

Compared to patients who received no or only one diuretic, patients who received two or more diuretics were more often hyponatremic (*χ*^*2*^ = 10.1;*p* = 0.006). Hyponatremic patients received spironolactone more often, as well as the combination of furosemide with spironolactone (Table 1). We observed no significant association of concomitant use of furosemide, hydrochlorothiazide and spironolactone in combination with hyponatremia (Table 1) probably due to the small number of such patients (*n* = 11).

**Fig. 1:**
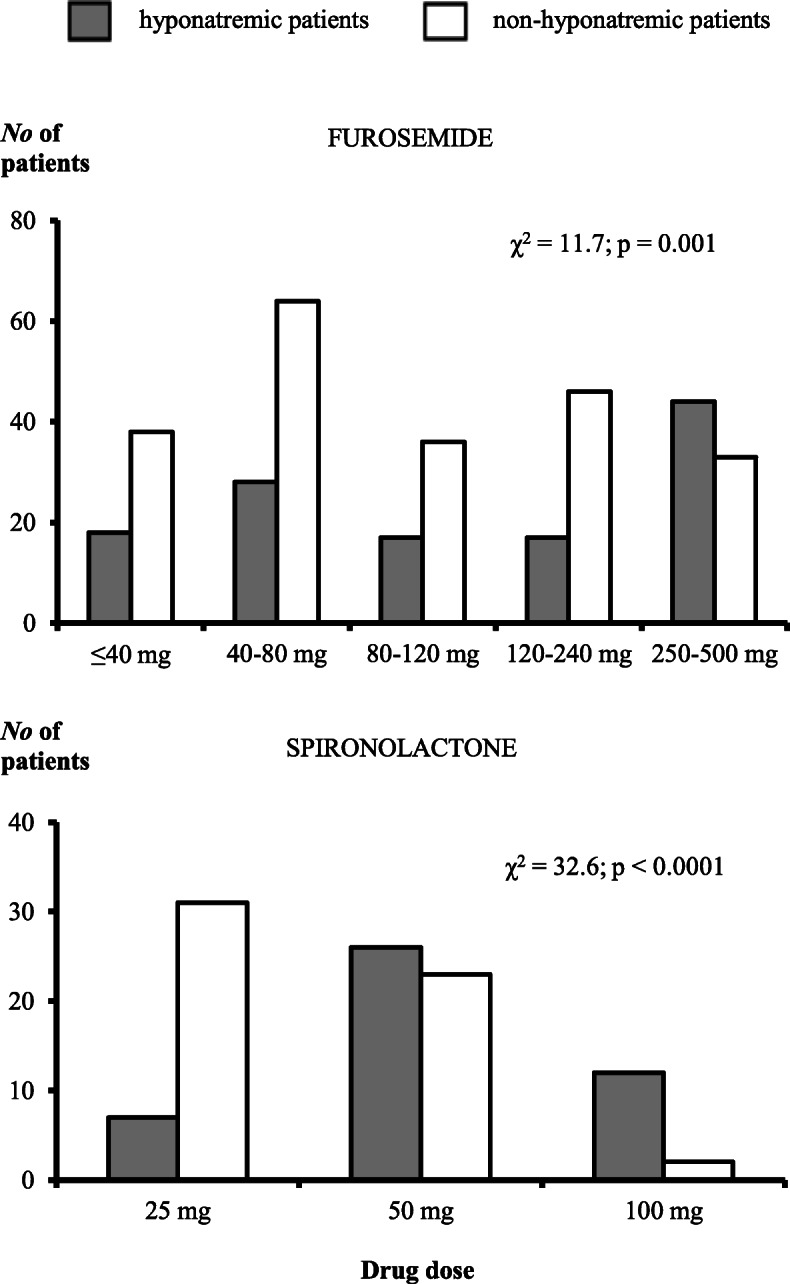
Distribution of study patients according to furosemide and spironolactone dose and hyponatremia

When considering drug dose, patients who received 50 or 100 mg of spironolactone were significantly more often hyponatremic than those receiving 25 mg, as were those receiving ≥250 mg of furosemide compared to those receiving lower doses (Fig. 1). Patients who received either 50 or 100 mg of spironolactone almost always concomitantly used furosemide while those receiving 25 mg more often used spironolactone only (Fig. S.1, Additional file 1). There was no significant association between dose of hydrochlorothiazide and hyponatremia (Fig. S.2, Additional file 1).

Patients who received calcium antagonists had a significantly higher LVEF compared to those who did not (54% vs 46%; *p* < 0.001). Since the collection of plasma level of NT-proBNP was not included in the study protocol, this data was available for 148 of our patients. The levels of this marker of HF severity were significantly higher in hyponatremic (*n* = 41) than in non-hyponatremic (*n* = 107) patients; 969.1 pmol/L (median, IQR 307.6–2004.0) vs. 423.4 pmol/L (IQR 195.5–977.3); *p* = 0.006. In the univariate analysis, NT-proBNP levels significantly correlated with the GFR, LVEF, and hydrochlorothiazide dose (Table S.1, Additional file 1).

### Multivariate analysis

Because of the interrelation among drug doses in combination with diuretics and hyponatremia, we ran three separate multivariate models. When we included the use of each diuretic as a dichotomous variable (Table S.2, Additional file 1), we found no significant association with hyponatremia. In the second model, a combination of furosemide and spironolactone was an independent predictor of hyponatremia (Table S.3, Additional file 1). In the last model, we stratified the patients according to the results of the univariate analysis with cut-off doses for furosemide and spironolactone. We observed a significant independent association of both furosemide doses of ≥250 mg and spironolactone doses ≥50 mg with hyponatremia (Table 3). In all the three models, increasing age, diabetes mellitus and alcohol consumption were independent predictors of hyponatremia. Low GFR, low LVEF, cardiac troponin I levels and both spironolactone use and its dose were independent predictors of NT-proBNP levels (Table S.4, Model 1 and 2, Additional file 1), after adjustment for baseline clinical characteristics, other markers of HF severity and cardiovascular drugs with possible impact on NT-proBNP levels.

**Table 3:** Multivariate analysis of the predicting association of clinical factors with occurrence of hyponatremia

	*OR* (95% *CI*)	*p*
Age (per 10-year increase)	1.115 (1.023–1.159)	0.03*
Alcohol consumption	1.112 (1.002–1.273)	0.04*
Male sex	0.947 (0.861–1.047)	0.29
Kidney failure	1.043 (0.945–1.154)	0.39
LVEF ≤45%	0.943 (0.994–1.001)	0.25
Arterial hypertension	0.947 (0.854–1.049)	0.29
Diabetes mellitus	1.114 (1.012–1.224)	0.02*
Previous AMI	1.053 (0.946–1.191)	0.31
Current smoking	1.069 (0.953–1.258)	0.19
Hydrochlorothiazide	0.976 (0.863–1.093)	0.63
250 to 500 mg furosemide	1.138 (1.043–1.344)	0.009*
50 to 100 mg spironolactone	1.197 (1.126–1.484)	0.0003*
β-blocker	0.959 (0.878–1.053)	0.39
Calcium antagonist	0.935 (0.827–1.036)	0.18
ARB	1.049 (0.929–1.249)	0.32
ACEI	1.074 (0.973–1.178)	0.16
Aspirin	0.977 (0.886–1.076)	0.63
Digoxin	0.943 (0.837–1.045)	0.24

Odds ratios (*OR*) and *p* values were obtained from the logistic regression analysis. *CI* Confidence Interval, *LVEF* Left ventricular ejection fraction, *AMI* Acute myocardial infarction, *ARB* Angiotensin II receptor I blocker, *ACEI* Angiotensin converting enzyme-inhibitor

* Statistically significant differences (*p* < 0.05)

## Discussion

When accounting for demographic and clinical variables, the doses of ≥250 mg of furosemide and ≥ 50 mg of spironolactone received for at least a month, or concomitant use of both diuretics regardless of their dose, were independent predictors of hyponatremia at hospital admission of patients due to HF. In addition, increasing age, diabetes mellitus and alcohol consumption were baseline characteristics independently associated with the occurrence of hyponatremia.

Hyponatremia is a predictor of adverse outcomes, in both short- and long-term prognosis [9, 10, 20–26], and has been closely associated with increased length of in-hospital stay [23, 24], rate of rehospitalizations, major complications [25], and risk for in-hospital mortality [24–26]. In addition, hyponatremia has been suggested as an important predictor of adverse in-hospital outcomes in all HF patients, regardless of the LVEF [27]. Our univariate analysis showed that hyponatremic patients more often had LVEF ≤45%, but this association disappeared in the multivariate analysis. In HF, hyponatremia is a product of several simultaneous pathophysiological mechanisms, including arterial underfilling, activation of the sympathetic nervous system and renin-angiotensin-aldosterone system, enhanced arginine vasopressin secretion, renal impairment and diuretic therapy [2, 6]. Our study suggests that the net-effect of these mechanisms is more important for the occurrence of hyponatremia than isolated effect of LVEF. An inverse association of LVEF and GFR with NT-proBNP levels in our patients is compatible to the basic mechanisms of failing heart within the pathophysiological framework of the cardiorenal syndrome [28].

In addition to some smaller studies [29–31], a large cohort study using data from the Rotterdam study [12] and a retrospective study by Arampatzis et al. [14] have reported the association of thiazide use with hyponatremia. In contrast, we did not confirm such an association, regardless of the hydrochlorothiazide dose. However, none of our HF patients took > 25 mg of hydrochlorothiazide. Most recently, the dose of ≥25 mg of hydrochlorothiazide, in addition to advanced age, female sex, use of benzodiazepines or statins and previous cerebrovascular accident, has been suggested as a clinical predictor of hyponatremia among hypertensive patients taking thiazide diuretics [32]. There is a possibility that metabolic and other effects of thiazide diuretics may vary in specific populations which should be accounted for in future studies.

Several studies have linked the use of loop diuretics to hyponatremia [15, 33]. Loop diuretics promote natriuresis and water loss through the inhibition of sodium chloride reabsorption at the ascending limb of Henle’s loop [17]. However, these drugs act at the macula densa, affecting both renal concentrating and diluting mechanisms [17], and when water loss is insufficiently replaced, hypernatremia may also occur [12, 33]. In contrast to some exceptions [9], our finding of 60% of HF patients taking furosemide is in agreement with other studies on this topic [15, 34]. We found a strong association of high doses, i.e., 250 to 500 mg of furosemide with hyponatremia, in both univariate and multivariate analysis. In the previous studies, loop diuretics were analyzed as the class, and dosage was not taken into account [9, 12, 14, 15, 33]. This may at least partly explain conflicting results in the association between loop diuretic use and serum sodium levels, including hyponatremia [15, 33], hypernatremia [12, 33] or lack of association [9, 14].

A recent study in stable HF patients, in whom the furosemide dose was reduced by 50%, has shown that this change may cause a 20% increase in GFR among those with GFR lower than 60 ml/min/1.73 m^2^ [35]. We observed no association of kidney failure with hyponatremia in either univariate or multivariate analysis. This suggests that other significant predictors such as diuretics, increasing age, diabetes mellitus or alcohol consumption play a more important role in the occurrence of hyponatremia. Lowering GFR may be an adverse effect of higher diuretic doses which occasionally may worsen kidney failure.

In this line, a reduction of furosemide dose of ≥120 mg to a third of the baseline dose in patients with HF, has been associated with increased 1- and 2-year survival rates free of hospitalization or cardiac death [36]. Our results extend this knowledge by strongly linking the dose of ≥250 mg to hyponatremia. Therefore, a careful titration and reduction of furosemide dose below 250 mg seems to be an important clinical goal, whenever possible.

Spironolactone, a potassium sparing diuretic, has also been implicated in the development of hyponatremia [10, 12]. A possibility that coadministration of a second diuretic with potassium sparing diuretic can either cause or aggravate hyponatremia has been reported [17, 18]. A fact that every fourth of our patients concomitantly used spironolactone with furosemide, and nearly all of those who took ≥50 mg of spironolactone, supports this possibility. When drug doses were taken into account, we observed an independent association of high spironolactone dose (50 or 100 mg daily) with hyponatremia. By inhibiting aldosterone-induced synthesis of epithelial Na^+^ channels and consequently, Na^+^-K^+^ exchange, spironolactone enhances natriuresis [17]. Our results suggest that dose of 25 mg of spironolactone is relatively safe, while doses of 50 or 100 mg, or concomitant use with furosemide, probably enhance natriuresis to the extent that often cause hyponatremia. As in the case of furosemide, this suggests that a reduction of the spironolactone dose may be a useful clinical approach. The strong positive association between NT-pro BNP levels and spironolactone use, as well as its dose, could be a consequence of more frequent spironolactone prescription in higher dose to patients with most severe clinical HF. Interestingly, the dose of furosemide did not correlate with NT-pro BNP levels. It may be assumed that introduction of spironolactone and increase in its dose is most often gradual and compatible with the severity of HF. In contrast, variations in the dose of furosemide could be more frequent and more subjected to other clinical symptoms associated with electrolyte disbalance, changes in volume status and other pathophysiological consequences of cardiorenal syndrome.

Hyponatremia commonly occurs in elderly patients [4–6] which is supported by our results. The underlying pathophysiological mechanisms include age-related impairment in water excretory capacity causing a reduction in GFR [4], concomitant use of diuretics and central nervous system active drugs [4, 5], the syndrome of inappropriate antidiuretic hormone secretion [37] and the presence of other comorbidities such as HF [1, 2], diabetes mellitus [7, 8], kidney failure [9], cirrhosis [6] and others [6]. Alcohol consumption was also independently associated with hyponatremia. Patients with a chronic alcohol-use disorder commonly exhibit hyponatremia, as a result of increased vasopressin levels which induce an increased urine osmolality and decreased clearance of free water [11].

Diabetic patients frequently develop a wide array of electrolyte and acid-base disorders [7, 8]. Patients with diabetes mellitus are usually hyponatremic [8], a finding which is concurred by our results. In the hyperglycemic state, hyponatremia develops as a result of transition of water out of the cells and a reduction in sodium levels through dilution [7]. On the other hand, increased or normal serum sodium levels in diabetic patients may be associated with a significant deficit in total body water [8]. Other important causes and types of sodium disorders in patients with diabetes include osmotic diuresis induced hypovolemic hyponatremia [38], drug induced hyponatremia (oral hypoglycemics, insulin, tricyclic antidepressants) [39], pseudohyponatremia induced by hypertriglyceridemia [40] and pseudohypernatremia due to severe hypoproteinemia [7].

Cardiac troponin is another biomarker with an important prognostic value in acute HF, which has been consistently shown to correlate with increased morbidity and mortality [41]. A positive association between NT-proBNP and troponin I levels observed in our patients confirms its role as a valuable clinical indicator of HF severity. Calcium antagonist therapy is not recommended in patients with HF and reduced LVEF [42]. Our patients who received calcium antagonists had a significantly higher and on average preserved LVEF which indicates that these medications are predominantly prescribed according to current recommendations.

### Study limitations

There are several limitations to our study. Firstly, in addition to serum sodium level, volemic status is a closely interrelated factor which determines the course and prognosis of HF patients. Both factors are significantly dependent on the effects of diuretic therapy. However, we did not have an insight into the patients’ volume status, but evaluation of congestion and clinical euvolaemia remains challenging since no reliable clinical test exists to determine euvolaemia [43]. Secondly, we did not collect data on several medications which may also influence serum sodium level, such as benzodiazepines, antiepileptics, selective serotonin reuptake inhibitors and non-steroidal anti-inflammatory drugs [38]. Thirdly, we did not collect data on the New York Heart Association class, previous HF hospitalizations, duration or worsening of HF symptoms, and mechanisms of death in our patients. Finally, we investigated only patients with HF, and there is a possibility that our findings may not be generalized to other patient subgroups.

## Conclusions

The pathogenesis of hyponatremia is multifactorial. Among patients hospitalized because of HF, doses of ≥250 mg of furosemide and ≥ 50 mg of spironolactone received for at least a month, or concomitant use of both diuretics regardless of their dose, were independently associated with hyponatremia at admission. Future research should explore whether a targeted reduction of dose of furosemide and spironolactone in clinically stable HF patients, particularly if they are elderly, diabetics or alcohol consumers, is safe and tolerable and whether this may help avoid hyponatremia and improve their clinical status and prognosis.

## Supplementary information

**Additional file 1: Fig. S.1.** Patients taking spironolactone according to dose and coadministration with furosemide. **Fig. S.2.** Distribution of study patients according to hydrochlorothiazide dose and hyponatremia. **Table S.1.** Univariate correlations of clinical variables with serum NT-proBNP levels. **Table S.2 (Model 1).** Multivariate analysis of the predicting association of clinical factors with occurrence of hyponatremia. **Table S.3 (Model 2).** Multivariate analysis of the predicting association of clinical factors with occurrence of hyponatremia. **Table S.4 (Model 1, Model 2).** Multivariate analysis of the predicting association of clinical factors with serum NT-proBNP levels.

## Data Availability

The datasets analyzed during the current study are available from the corresponding author on reasonable request.

## Abbreviations

HF: Heart failure
LVEF: Left ventricular ejection fraction
NT-proBNP: N-terminal pro brain natriuretic peptide
GFR: Glomerular filtration rate
IQR: Interquartile range
SPSS: Statistical package for the social science
