# Supplementary material for: Furosemide and spironolactone doses and hyponatremia in patients with heart failure
Source: BMC Pharmacol Toxicol. 2020 Aug 3;21:57. doi: 10.1186/s40360-020-00431-4 (PMC7397681; doi:10.1186/s40360-020-00431-4)
Supplement: Supplementary file 1 — Additional file 1: Fig. S.1. Patients taking spironolactone according to dose and coadministration with furosemide. Fig. S.2. Distribution of study patients according to hydrochlorothiazide dose and hyponatremia. Table S.1. Univariate correlations of clinical variables with serum NT-proBNP levels. Table S.2 (Model 1). Multivariate analysis of the predicting association of clinical factors with occurrence of hyponatremia. Table S.3 (Model 2). Multivariate analysis of the predicting association of clinical factors with occurrence of hyponatremia. Table S.4 (Model 1, Model 2). Multivariate analysis of the predicting association of clinical factors with serum NT-proBNP levels. [file 40360_2020_431_MOESM1_ESM.docx]

**Furosemide and spironolactone doses and hyponatremia in patients with heart failure**

**Supplementary material**

**Authors:** Ivan Velat, Željko Bušić, Marina Jurić Paić, Viktor Čulić

**Journal name:** BMC Pharmacology and Toxicology

**Correspondence:** Prof. Viktor Čulić

Department of Cardiology

University Hospital Center Split

Šoltanska 1

21000 Split, Croatia

Tel: ++ 385 21 55 72 89

Fax: ++ 385 21 55 73 85

E-mail: [viktor.culic@st.t-com.hr](mailto:viktor.culic@st.t-com.hr)

This supplement has been provided by the authors to give readers additional information about the findings of the study.

1. **Fig. S.1** Patients taking spironolactone according to dose and coadministration with furosemide
2. **Fig. S.2** Distribution of study patients according to hydrochlorothiazide dose and hyponatremia
3. **Table S.1** Univariate correlations of clinical variables with serum NT-proBNP levels.
4. **Table S.2 (Model 1)** Multivariate analysis of the predicting association of clinical factors with occurrence of hyponatremia
5. **Table S.3 (Model 2)** Multivariate analysis of the predicting association of clinical factors with occurrence of hyponatremia
6. **Table S.4**. **(Model 1, Model 2)** Multivariate analysis of the predicting association of clinical factors with serum NT-proBNP levels

**Fig. S.1**

**Fig. S.2**

**Table S.1**

Univariate correlations of clinical variables with serum NT-proBNP levels.

|  | *r* | *p* |
| --- | --- | --- |
| GFR (ml/min/1.73 m^2^) | 0.164 | 0.047* |
| Cardiac troponin I (ng/ml) | 0.039 | 0.67 |
| Furosemide dose (mg) | -0.032 | 0.70 |
| Hydrochlorothiazide dose (mg) | 0.276 | 0.001* |
| Spironolactone dose (mg) | 0.021 | 0.80 |
| LVEF (%) | -0.313 | <0.001* |
| Serum sodium (mmol/L) | -0.049 | 0.55 |

*NT-pro BNP*: N-terminal pro brain natriuretic peptide, *GFR*: glomerular filtration rate, *LVEF*: left ventricular ejection fraction.

*r* and *p* values were obtained from the linear regression analysis.

* Statistically significant differences (*p*<0.05)

**Table S.2**

Multivariate analysis of the predicting association of clinical factors with occurrence of hyponatremia (Model 1*)

|  | *OR* (95% *CI*) | *p* | |
| --- | --- | --- | --- |
| Age (per 10-year increase) | 1.116 (1.009 – 1.237) | | 0.03^†^ |
| Alcohol consumption | 1.113 (1.005 – 1.282) | | 0.04^†^ |
| Male sex | 0.952 (0.865 – 1.052) | | 0.35 |
| Kidney failure | 1.044 (0.940 – 1.165) | | 0.40 |
| LVEF ≤ 45% | 1.080 (0.978 – 1.185) | | 0.13 |
| Arterial hypertension | 0.932 (0.838 – 1.033) | | 0.18 |
| Diabetes mellitus | 1.119 (1.015 – 1.232) | | 0.02^†^ |
| Previous AMI | 1.068 (0.962 – 1.213) | | 0.19 |
| Current smoking | 1.066 (0.947 – 1.256) | | 0.23 |
| Furosemide | 1.022 (0.919 – 1.134) | | 0.69 |
| Hydrochlorothiazide | 0.957 (0.840 – 1.071) | | 0.39 |
| Spironolactone | 1.079 (0.971 – 1.247) | | 0.13 |
| β-blocker | 0.979 (0.894 – 1.075) | | 0.67 |
| Calcium antagonist | 0.918 (0.824 – 1.021) | | 0.12 |
| ARB | 1.047 (0.923 – 1.247) | | 0.36 |
| ACEI | 1.069 (0.967 – 1.176) | | 0.19 |
| Aspirin | 0.968 (0.876 – 1.067) | | 0.50 |
| Digoxin | 0.960 (0.852 – 1.071) | | 0.43 |

Odds ratios (*OR*) and *p* values were obtained from the logistic regression analysis. *CI*: Confidence Interval, *LVEF*: left ventricular ejection fraction, *AMI*: acute myocardial infarction, *ARB*: angiotensin II receptor I blocker, *ACEI*: angiotensin converting enzyme-inhibitor

* The use of each drug was included as dichotomous variable

^†^ Statistically significant differences (*p*<0.05)

**Table S.3**

Multivariate analysis of the predicting association of clinical factors with occurrence of hyponatremia (Model 2*)

|  | *OR* (95% *CI*) | *p* |
| --- | --- | --- |
| Age (per 10-year increase) | 1.115 (1.006 – 1.239) | 0.03^†^ |
| Alcohol consumption | 1.114 (1.007 – 1.146) | 0.03^†^ |
| Male sex | 0.939 (0.853 – 1.041) | 0.24 |
| Kidney failure | 1.043 (0.943 – 1.158) | 0.39 |
| LVEF ≤ 45% | 0.945 (0.994 – 1.001) | 0.27 |
| Arterial hypertension | 0.934 (0.841 – 1.035) | 0.19 |
| Diabetes mellitus | 1.109 (1.003 – 1.224) | 0.04^†^ |
| Previous AMI | 1.057 (0.949 – 1.198) | 0.28 |
| Current smoking | 1.066 (0.947 – 1.255) | 0.22 |
| Furosemide + spironolactone | 1.113 (1.007 – 1.272) | 0.04^†^ |
| Hydrochlorothiazide | 0.930 (0.810 – 1.036) | 0.16 |
| β-blocker | 0.984 (0.899 – 1.079) | 0.75 |
| Calcium antagonist | 0.923 (0.814 – 1.023) | 0.12 |
| ARB | 1.053 (0.931 – 1.258) | 0.30 |
| ACEI | 1.067 (0.966 – 1.173) | 0.21 |
| Aspirin | 0.973 (0.881 – 1.073) | 0.58 |
| Digoxin | 0.955 (0.848 – 1.062) | 0.36 |

Odds ratios (*OR*) and *p* values were obtained from the logistic regression analysis. *CI*: Confidence Interval, *LVEF*: left ventricular ejection fraction, *AMI*: acute myocardial infarction, *ARB*: angiotensin II receptor I blocker, *ACEI*: angiotensin converting enzyme-inhibitor

* The use of each drug, as well as combination of furosemide and spironolactone, were included as

dichotomous variable

^†^ Statistically significant differences (*p*<0.05)

**Table S.4**

Multivariate analysis of the predicting association of clinical factors with serum NT-proBNP levels.

|  | Model 1* |  |  | Model 2** |  |
| --- | --- | --- | --- | --- | --- |
|  | *β* | *p* |  | *β* | *p* |
| Predictors |  |  |  |  |  |
| Age (years) | 0.078 | 0.39 |  | 0.095 | 0.32 |
| Alcohol consumption | 0.034 | 0.69 |  | 0.030 | 0.73 |
| Male sex | -0.104 | 0.24 |  | -0.049 | 0.59 |
| GFR (ml/min/1.73 m^2^) | -0.458 | <0.0001^†^ |  | -0.492 | <0.0001^†^ |
| LVEF (%) | -0.173 | 0.04^†^ |  | -0.193 | 0.02^†^ |
| Cardiac troponin I (ng/ml) | 0.177 | 0.04^†^ |  | 0.178 | 0.04^†^ |
| Arterial hypertension | 0.051 | 0.58 |  | 0.077 | 0.42 |
| Diabetes mellitus | 0.057 | 0.52 |  | 0.027 | 0.76 |
| Previous AMI | -0.002 | 0.98 |  | 0.008 | 0.93 |
| Current smoking | 0.031 | 0.71 |  | 0.052 | 0.54 |
| Furosemide | 0.037 | 0.71 |  | 0.006 | 0.95 |
| Spironolactone | 0.312 | <0.0001^†^ |  | 0.275 | 0.003^†^ |
| Hydrochlorothiazide | 0.035 | 0.67 |  | 0.064 | 0.44 |
| Calcium antagonist | -0.130 | 0.13 |  | -0.129 | 0.15 |
| ARB | -0.168 | 0.053 |  | -0.173 | 0.051 |
| ACEI | -0.096 | 0.27 |  | -0.090 | 0.31 |
| Digoxin | -0.079 | 0.38 |  | -0.055 | 0.53 |

*NT-pro BNP*: N-terminal pro brain natriuretic peptide, *GFR*: glomerular filtration rate, *LVEF*: left ventricular ejection fraction, *AMI*: acute myocardial infarction*, ARB*: angiotensin II receptor I blocker, *ACEI*: angiotensin converting enzyme-inhibitor. *β* and *p* values were obtained from the multiple regression analysis

* Model 1 includes furosemide, spironolactone and hydrochlorothiazide as dichotomous variables

** Model 2 includes furosemide, spironolactone and hydrochlorothiazide with their doses (mg)

^†^ Statistically significant differences (*p*<0.05)
